# Practical investigation of the performance of robust logistic regression to predict the genetic risk of hypertension

**DOI:** 10.1186/1753-6561-8-S1-S65

**Published:** 2014-06-17

**Authors:** Miriam Kesselmeier, Carine Legrand, Barbara Peil, Maria Kabisch, Christine Fischer, Ute Hamann, Justo Lorenzo Bermejo

**Affiliations:** 1 Institute of Medical Biometry and Informatics, University of Heidelberg, Im Neuenheimer Feld 305, 69120 Heidelberg, Germany; 2 Molecular Genetics of Breast Cancer, Deutsches Krebsforschungszentrum (DFKZ), Im Neuenheimer Feld 580, 69120 Heidelberg, Germany; 3 Institute of Human Genetics, University Hospital Heidelberg, Im Neuenheimer Feld 366, 69120 Heidelberg, Germany; 4 Clinical Epidemiology, Integrated Research and Treatment Center, Center for Sepsis Control and Care (CSCC), Jena University Hospital, Erlanger Allee 101, 07747 Jena, Germany

## Abstract

Logistic regression is usually applied to investigate the association between inherited genetic variants and a binary disease phenotype. A limitation of standard methods used to estimate the parameters of logistic regression models is their strong dependence on a few observations deviating from the majority of the data.

We used data from the Genetic Analysis Workshop 18 to explore the possible benefit of robust logistic regression to estimate the genetic risk of hypertension. The comparison between standard and robust methods relied on the influence of departing hypertension profiles (outliers) on the estimated odds ratios, areas under the receiver operating characteristic curves, and clinical net benefit.

Our results confirmed that single outliers may substantially affect the estimated genotype relative risks. The ranking of variants by probability values was different in standard and in robust logistic regression. For cutoff probabilities between 0.2 and 0.6, the clinical net benefit estimated by leave-one-out cross-validation in the investigated sample was slightly larger under robust regression, but the overall area under the receiver operating characteristic curve was larger for standard logistic regression. The potential advantage of robust statistics in the context of genetic association studies should be investigated in future analyses based on real and simulated data.

## Background

Hypertension is a common chronic medical condition characterized by elevated arterial blood pressure. High blood pressure is associated with an increased risk of stroke, heart attack, and other serious diseases. Age, gender, tobacco smoking, alcohol consumption, and high body mass index constitute established risk factors for hypertension [1]. A genetic component has also been postulated. It has been shown that individuals with a family history of hypertension have on average a higher blood pressure than individuals without a family history. Yanek et al found a 44% higher prevalence of hypertension in siblings of affected persons than in the general reference population [2]. In a Canadian study, standardized risk ratios of hypertension were higher for first-degree relatives than for spouses of probands with hypertension [3]. In genetic studies, a large number of polymorphisms has been associated with hypertension and validated in independent collectives; 14 loci have been identified (as of 2010) and many genetic studies are currently in progress [4-8].

The relationship between inherited genetic polymorphisms and a binary response variable (with/without hypertension) can be investigated using logistic regression models that simultaneously consider the effects of multiple risk factors. Standard methods used to estimate the parameters of logistic regression models--for example, iteratively reweighted least squares--are limited by their dependence on a few observations departing from the majority of the data. This contrasts with the purpose of genetic risk models that aim to predict a particular health outcome that holds for the bulk of individuals, and to identify persons with a deviating high risk of disease. We use data from the Genetic Analysis Workshop (GAW18) to explore the possible benefit of robust parameter estimates in logistic regression models for the genetic prediction of hypertension risk.

## Methods

The analysed data (real phenotypes) were derived from 142 unrelated individuals who participated in the San Antonio Family Heart or Family Diabetes/Gallbladder studies. Longitudinal information on hypertension, age, gender, and current tobacco smoking was measured up to 4 times per individual; the present analyses relied on the first available measurement. Further information is provided in several articles [9-12].

The original data was filtered according to the following criteria: (a) at least 1 measurement with complete information on hypertension and age, (b) monomorphisms were excluded and each polymorphism had to be represented by at least 2 individuals, (c) individuals with more than 5% missing genotypes were excluded, and, finally, (d) variants with missing data in any individual were removed.

The relationship between hypertension and age, gender, and current tobacco smoking was first investigated by χ^2 ^tests. Covariates significantly associated at the 5% confidence level entered the intercept-only model to build the baseline model. Subsequently, standard logistic regression (iteratively reweighted least squares) was used to identify possible hypertension-associated single-nucleotide polymorphisms (SNPs) with minimal deviance, taking into account associated covariates. The deviance is defined as minus twice the logarithm of the likelihood. Genotypes were coded according to an additive penetrance model; that is, 0, 1, and 2. Departing observations (outliers) according to standard logistic regression were identified based on the Cook's distance in the baseline model. The Cook's distance for observation i is defined as

$${\text{D}}_{\text{i}}=\frac{\sum_{\text{j}=1}^{\text{n}}{\left({\hat{\text{y}}}_{\text{j}}-{\hat{\text{y}}}_{\text{j}\left(\text{i}\right)}\right)}^{2}}{\text{q}\;\text{MSE}}$$

where ${\hat{\text{y}}}_{\text{j}}$ denotes the full regression model prediction for observation j, ${\hat{\text{y}}}_{\text{j}\left(\text{i}\right)}$ represents the regression model prediction for observation j estimated omitting observation i, and MSE indicates the mean square error of the regression model with *q *explanatory variables.

To investigate the possible benefit of robust parameter estimates in logistic regression, model coefficients were also estimated by solving

$$\overset{\text{n}}{\underset{\text{i}=1}{\sum }}\Psi \left({\text{y}}_{\text{i}};\mu_{\text{i}}\right)=\overset{\text{n}}{\underset{\text{i}=1}{\sum }}\text{v}\left({\text{y}}_{\text{i}};\mu_{\text{i}}\right)\text{w}\left({\text{x}}_{\text{i}}\right){\mu_{\text{i}}}^{\prime }-\alpha \left(\beta \right)=0$$

where $\text{v}\left({\text{y}}_{\text{i}};\mu_{\text{i}}\right)=\frac{\psi_{\text{c}}\left(\epsilon_{\text{i}}\right)}{{\text{V}}^{1/2}\left(\mu_{\text{i}}\right)}$ with the Pearson residuals $\epsilon_{\text{i}}$ and the Huber function

$\psi_{\text{c}}\left({\text{r}}_{\text{i}}\right)=\left\{\begin{array}{cc} {\text{r}}_{\text{i}} & \text{for}\;\left|{\text{r}}_{\text{i}}\right|\leq \text{c}\\ \text{c}\;\text{sign}\left({\text{r}}_{\text{i}}\right) & \text{for}\;\left|{\text{r}}_{\text{i}}\right|>c, \end{array}\right)$$w\left(x_{i}\right)={\left(1-h_{ii}\right)}^{1/2}$ with $h_{ii}$ the i^th ^diagonal element of the matrix $H=X{\left(X^{T}X\right)}^{-1}X^{T}$, $\mu_{\text{i}}^{\text{'}}=\frac{\partial \mu_{\text{i}}}{\partial \beta }$ and $\alpha \left(\beta \right)=\frac{1}{2}\overset{\text{n}}{\underset{\text{i}=1}{\sum }}\text{E}\left[\text{v}\left({\text{y}}_{\text{i}};\;\mu_{\text{i}}\right)\right]\text{w}\left({\text{x}}_{\text{i}}\right)\mu_{\text{i}}^{\text{'}}$.

This estimator is based on a quasi-likelihood, asymptotically normally distributed and Fisher consistent [13]. The objective of the Huber function is to downweight the influence of outliers and to assign inliers the usual weight. Variable selection under robust logistic regression relied on the minimal quasideviance as described by Cantoni and Ronchetti, which is a robust test statistic for model selection [13]. The quasideviance between 2 nested models is defined as

$$\Lambda_{QM}=2\left[\overset{n}{\underset{i=1}{\sum }}Q_{M}\left(y_{i},{\hat{\mu }}_{i}\right)-\overset{n}{\underset{i=1}{\sum }}Q_{M}\left(y_{i},{\dot{\mu }}_{i}\right)\right]$$

where $Q_{M}\left(y_{i},\mu_{i}\right)=\int_{\tilde{s}}^{\mu_{i}}v\left(y_{i},t\right)w\left(x_{i}\right)dt-\frac{1}{n}\sum_{j=1}^{n}\int_{\tilde{t}}^{\mu_{j}}E\left[v\left(y_{j},t\right)w\left(x_{j}\right)\right]dt$ with $\tilde{s}$ such that $v\left(y_{i},\tilde{s}\right)=0$ and $\tilde{t}$ such that $E\left[v\left(y_{i},\tilde{t}\right)\right]=0$ and the estimated linear predictor $\hat{\mu }$ is associated to the estimate $\hat{\beta }$ of *β *and $\dot{\mu }$ is associated to $\dot{\beta }$ which is the estimate of $\left(\beta_{\left(1\right)},0\right)$. Linkage disequilibrium was not accounted for during variant selection neither for standard logistic regression nor for robust logistic regression.

Our comparison of the performance of standard and robust logistic regression was based on different statistics. First, standard and robust estimates of age effects were used to exemplify the potential influence of departing observations. Because of a different handling of outliers, it was expected that different age-genotype models were selected under standard and robust logistic regression. Consequently, the areas under the receiver operating characteristic curves (AUCs) were subsequently compared in order to investigate the discriminative performance of the selected models. Comparisons were conducted for the complete data set and after exclusion of potential outliers.

In addition, concordance, sensitivity, specificity, clinical net benefit, and AUCs were estimated for age-genotype models using a leave-one-out cross-validation approach [14]. Concordance was defined as the proportion of correctly estimated hypertension statuses using several cutoff values for the predicted affection probability. The clinical net benefit (NB) was defined by

$$\begin{array}{cc} \text{NB}\left(\text{c}\right) & =\frac{\text{True}\;\text{positive}\;\text{counts}}{\text{Sample}\;\text{size}}-\frac{\text{False}\;\text{positive}\;\text{counts}}{\text{Sample}\;\text{size}}\cdot \frac{\text{c}}{1-\text{c}}\\ & =\text{Sensitivity}\cdot \left(\text{\% }\;\text{Hypertensive}\right)-\left(1-\text{Specificity}\right)\cdot \left(\text{\% }\;\text{Normotensive}\right)\cdot \frac{\text{c}}{1-\text{c}} \end{array}$$

where *c *is the chosen threshold for allocating an individual to the cases based on the logistic regression probability estimate. Note that the net benefit depends on the hypertension prevalence in the study population. The standard and robust logistic regression models were also compared based on the integrated discrimination index (IDI) estimated by cross-validation

$$\text{IDI}=\left(\frac{1}{{\text{n}}_{\text{cases}}}\overset{{\text{n}}_{\text{cases}}}{\underset{\text{i}=1}{\sum }}{\hat{\text{p}}}_{\text{rob},\;\text{i}}-\frac{1}{{\text{n}}_{\text{contr}}}\overset{{\text{n}}_{\text{contr}}}{\underset{\text{j}=1}{\sum }}{\hat{\text{p}}}_{\text{rob},\;\text{j}}\right)\;-\left(\frac{1}{{\text{n}}_{\text{cases}}}\overset{{\text{n}}_{\text{cases}}}{\underset{\text{i}=1}{\sum }}{\hat{\text{p}}}_{\text{stand},\;\text{i}}-\frac{1}{{\text{n}}_{\text{contr}}}\overset{{\text{n}}_{\text{contr}}}{\underset{\text{j}=1}{\sum }}{\hat{\text{p}}}_{\text{stand},\;\text{j}}\right)$$

where ${\hat{\text{p}}}_{\text{rob},\;\text{i}}$, ${\hat{\text{p}}}_{\text{rob},\;\text{j}}$, ${\hat{\text{p}}}_{\text{stand},\;\text{i}}$, and ${\hat{\text{p}}}_{\text{stand},\;\text{j}}$ denote the probability estimates from the robust and standard logistic regression models for cases and controls [15]. This index represents the difference in the discrimination slopes of the 2 compared models. A positive IDI indicates that the robust model discriminates better between hypertensive and normotensive individuals than the standard model. Statistical analyses were carried out using the statistical language R, version 2.15.1 [16].

## Results

χ^2 ^tests revealed no influence of gender (*p *= 0.95) and tobacco smoking (*p *= 1.00) on hypertension risk. Hence, only age was included in the logistic regression models as covariate. Filter criteria resulted in 130 individuals (43 cases and 87 controls) with complete genotype and phenotype information. The age of the individuals ranged between 20 and 95 years with a median age of 52 years. The total number of measured SNPs on chromosome 3 in the investigated GAW18 data set was 35,045.

**Figure 1:**
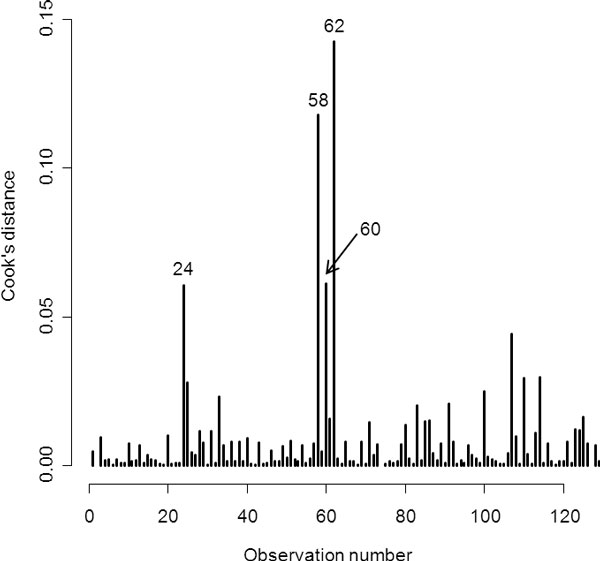
**Cook's distances from the age-only standard logistic regression model**. The 4 most prominent outliers are indicated by their observation number.

A plot of Cook's distances under the age-only standard logistic regression model revealed several observations (Figure 1) that departed from the majority of the sample. Considering a threshold of 0.05 for the Cook's distance, 4 observations could be defined as outliers. Information on disease status and age of deviating individuals is shown in Table 1. Individuals 62, 58, and 24 were older than 80 years and normotensive. Individual number 60 was affected by the condition early in life, at 38 years of age. Table 1 shows the influence of the 4 identified outliers on standard and robust parameter estimates of age effects. For example, the exclusion of individual 62 resulted in an 11.2% increase of the excess risk of hypertension per year according to standard logistic regression, compared to a 7.8% increase for robust logistic regression. Table 2 shows the odds of hypertension by age interval.

**Table 1:** Estimated odds ratios per year of age

Excluded individuals	HTN	Age	Standard logistic regression		Robust logistic regression	
				
			OR-Age (95% CI)	% Change	OR-Age (95% CI)	% Change
None			1.085 (1.050, 1.121)	ref.	1.084 (1.048, 1.122)	ref.
62	0	90.23	1.095 (1.057, 1.133)	+11.2%	1.091 (1.052, 1.131)	+7.8%
58	0	87.66	1.094 (1.056, 1.132)	+10.0%	1.091 (1.052, 1.131)	+7.9%
60	1	38.44	1.091 (1.054, 1.128)	+6.5%	1.089 (1.051, 1.128)	+5.1%
24	0	80.27	1.091 (1.054, 1.128)	+6.6%	1.091 (1.052, 1.131)	+7.6%

Odds ratios (ORs) were estimated based on standard and robust logistic regression models for the complete set of individuals and after exclusion of the 4 most remarkable outliers.

HTN: Hypertension.

**Table 2:** Overall odds of hypertension per age interval

**Age interval (number of cases-to-controls**)			
<39.0 (1:22)	[39.0, 46.0) (2:20)	[46.0, 56.2) (9:23)	≥56.2 (31:22)
0.05	0.10	0.39	1.41

Age intervals were defined by the age quartiles in controls.

**Figure 2:**
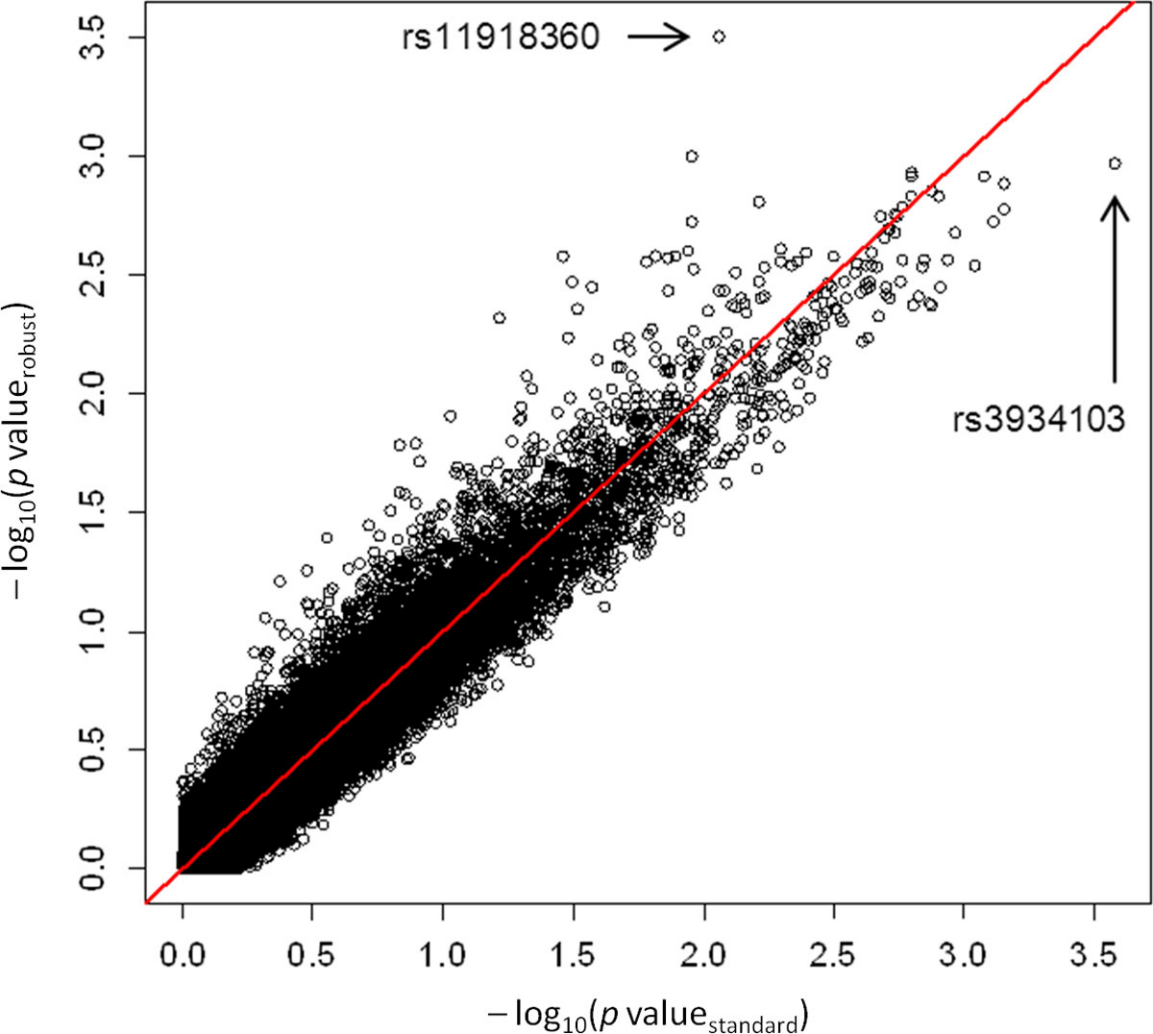
**Quantile-quantile plots from the age-genotype standard and robust logistic regression models**. The 2 selected SNPs are indicated by their reference SNP ID number.

Standard logistic regression identified SNP rs3934103 located in the *ULK4 *gene as the variant that most improved the model fit. Robust logistic regression identified SNP rs11918360 in *RP11-408H1.3 *as the variant with the strongest association signal. Under both standard and robust regression, model selection clearly favored the 2 identified SNPs as represented in Figure 2. The pairwise *r^2 ^*between SNP rs3934103 and SNP rs11918360 was 0.003.

Table 3 shows the influence of the 4 outliers on the AUCs from the standard and robust logistic regression models. Robust and standard AUCs for the age-only models were identical. For the age-genotype models, the AUCs were slightly smaller and also slightly less outlier-dependent for robust logistic regression than for standard logistic regression.

**Table 3:** Area under the receiver operating characteristic curve (AUC)

Excluded individuals	Standard logistic regression				Robust logistic regression			
		
	AUC-Age (% Change)		AUC-Age + SNP (% Change)		AUC-Age (% Change)		AUC-Age + SNP (% Change)	
None	0.811	(ref.)	0.852	(ref.)	0.811	(ref.)	0.843	(ref.)
62	0.820	+1.1%	0.861	+1.1%	0.820	+1.1%	0.852	+1.0%
58	0.820	+1.1%	0.861	+1.1%	0.820	+1.1%	0.853	+1.2%
60	0.825	+1.7%	0.859	+0.9%	0.825	+1.7%	0.851	+0.9%
24	0.819	+1.0%	0.859	+0.9%	0.819	+1.0%	0.844	+0.0%

AUCs were calculated for the complete set of individuals and after exclusion of the 4 most remarkable outliers. The relative contributions of the variables age and SNP (rs3934103 and rs11918360, respectively) are also shown.

Table 4 summarizes the results from the leave-one-out cross-validation. The concordance was better for the robust logistic regression model at every cutoff probability. Both models allocated best at probability 0.5 and almost identically at probability 0.3 (the investigated population included 43 cases and 87 controls; that is 33% hypertension prevalence). At a probability of 0.3, sensitivities were identical and the specificity was slightly higher under robust regression. Standard and robust estimates showed similar discriminative performances supported by an IDI of −0.07 at every cutoff probability. AUCs were also almost identical. The clinical net benefit was slightly larger for the robust logistic regression model in the probability range between 0.2 and 0.6.

**Table 4:** Concordance, sensitivity, specificity, clinical net benefit, and overall AUCs.

Probability cutoff	Standard logistic regression				Robust logistic regression			
		
	Concordance N (%)	Sensitivity	Specificity	Clinical net benefit	Concordance N (%)	Sensitivity	Specificity	Clinical net benefit
0.0	43 (33.1)	1.00	0.00	0.33	43 (33.1)	1.00	0.00	0.33
0.1	79 (60.8)	0.95	0.44	0.27	82 (63.1)	0.88	0.51	0.26
0.2	90 (69.2)	0.86	0.61	0.22	97 (74.6)	0.86	0.69	0.23
0.3	98 (75.4)	0.81	0.72	0.19	99 (76.2)	0.81	0.74	0.19
0.4	98 (75.4)	0.70	0.78	0.13	102 (78.5)	0.72	0.82	0.16
0.5	101 (77.7)	0.60	0.86	0.11	107 (82.3)	0.67	0.90	0.15
0.6	97 (74.6)	0.40	0.92	0.05	102 (78.5)	0.51	0.92	0.09
0.7	99 (76.2)	0.35	0.97	0.06	100 (76.9)	0.42	0.94	0.05
0.8	93 (71.5)	0.19	0.98	0.00	97 (74.6)	0.30	0.97	0.01
0.9	91 (70.0)	0.12	0.99	−0.03	93 (71.5)	0.19	0.98	−0.08
1.0	87 (66.9)	0.00	1.00	-	87 (66.9)	0.00	1.00	-
	
AUC	0.835				0.830			

These characteristics rely on the age-genotype models for standard and robust logistic regression estimated based on leave-one-out cross-validation.

## Discussion

Present results confirmed that single individuals (1/130 = 0.8% of the observations) with a departing risk of hypertension may substantially affect the overall risk estimates in the baseline model, causing up to an 11.2% change in the estimated excess risk of hypertension per year according to standard logistic regression in the present exercise.

The identification of outliers is relatively straightforward using routine diagnostic plots, but outlier management is extremely challenging. For example, the specification of thresholds for outlier definition is often arbitrary. Robust statistics aim to generate estimates that hold for the majority of the population using complete data. The unequal weighting of outliers by standard and robust regression resulted in prediction models that included different genetic variants.

Although robust estimates of age effects and AUCs for age-genotype models were less sensitive to outliers than standard estimates in the investigated sample, cross-validation AUCs based on standard and robust logistic regression, as well as IDI, were almost identical. The other investigated performance characteristics (concordance, sensitivity, specificity, and clinical net benefit) were equal or better for robust logistic regression around the probability that reflects the case-control ratio.

The standard logistic regression model selected 1 variant in the *ULK4 *gene. It was previously shown that variants in this gene are associated with hypertension [4,17]. Among others, 4 variants (rs2272007, rs3774372, rs1716975, rs1052501) mentioned in the 2 publications were also genotyped in the GAW18 collective, and we found them to be in linkage disequilibrium (*r^2 ^*values 0.83, 0.73, 0.83, and 0.83, respectively) with the associated SNP rs3934103.

## Conclusions

Preliminary findings suggest some advantage of robust statistics in the context of genetic association studies. However, present results were limited to a given sample size, as well as to particular genetic effect sizes and proportions of outliers. Additional analyses based on both real data and more general simulated scenarios should be conducted to validate initial findings.

## Competing interests

The authors declare that they have no competing interests.

## Authors' contributions

MK analysed and interpreted the data, created figures and tables, searched literature, and drafted the manuscript. CL identified relevant literature, supported data analysis and interpretation, and reviewed the manuscript. BP and MKa supported data analysis and interpretation and reviewed the manuscript. CF and UH supported interpretation and reviewed the manuscript. JLB formulated study goals, supported data analysis and interpretation, and reviewed the manuscript. All authors read and approved the final version.
